# Dual Attention-Based Industrial Surface Defect Detection with Consistency Loss

**DOI:** 10.3390/s22145141

**Published:** 2022-07-08

**Authors:** Xuyang Li, Yu Zheng, Bei Chen, Enrang Zheng

**Affiliations:** 1 School of Electrical and Control Engineering, Shaanxi University of Science and Technology, Xi’an 710021, China; 200611021@sust.edu.cn (X.L.); chenbei@sust.edu.cn (B.C.); 2 School of Cyber Engineering, Xidian University, Xi’an 710126, China; yuzheng.xidian@gmail.com

**Keywords:** surface defect detection, anomaly detection, industrial security, attention mechanism

## Abstract

In industrial production, flaws and defects inevitably appear on surfaces, resulting in unqualified products. Therefore, surface defect detection plays a key role in ensuring industrial product quality and maintaining industrial production lines. However, surface defects on different products have different manifestations, so it is difficult to regard all defective products as being within one category that has common characteristics. Defective products are also often rare in industrial production, making it difficult to collect enough samples. Therefore, it is appropriate to view the surface defect detection problem as a semi-supervised anomaly detection problem. In this paper, we propose an anomaly detection method that is based on dual attention and consistency loss to accomplish the task of surface defect detection. At the reconstruction stage, we employed both channel attention and pixel attention so that the network could learn more robust normal image reconstruction, which could in turn help to separate images of defects from defect-free images. Moreover, we proposed a consistency loss function that could exploit the differences between the multiple modalities of the images to improve the performance of the anomaly detection. Our experimental results showed that the proposed method could achieve a superior performance compared to the existing anomaly detection-based methods using the Magnetic Tile and MVTec AD datasets.

## 1. Introduction

**Figure 1:**
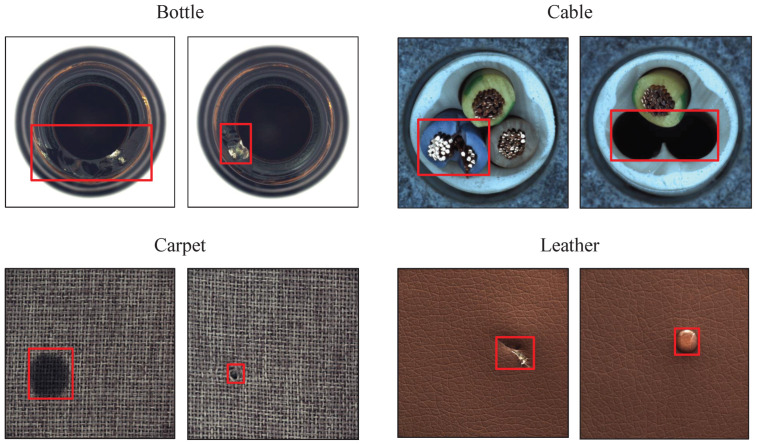
Industrial product samples with surface defects: each sub-figure represents a different industrial product (four in total) and each picture represents a different defect. The area in the red box contains the surface defect of each product.

Over recent years, surface defect detection has attracted attention in various fields, such as transportation [1,2,3], agriculture [4,5] and biomedicine [6,7], but surface defect detection has been especially extensively studied within manufacturing [8,9,10,11,12]. The process of industrial production is often accompanied by quality problems among the manufactured products and not all products can be monitored for quality through appearance observation. Therefore, surface defect detection plays an important role in industrial production. However, the surface defect detection of industrial products suffers from two main problems. First, a lack of defect instances: defective samples are usually rare among industrial products, while normal samples are common. Thus, it is difficult to collect enough defective samples and in extreme cases, only normal samples can be obtained. Second, the diverse types of defects, as shown in Figure 1: there are various types of defects among industrial products and the appearance of defects is not necessarily uniform on the same product. As a result, it is difficult to treat all defective products as one valid category. Under these circumstances, it is more appropriate to view the surface defect detection problem as a semi-supervised anomaly detection problem.

Anomaly detection has been widely used in various fields, including cyber security [13,14], communications security [15,16,17], IoT [18,19,20], video surveillance [21,22], etc. In general, anomaly detection refers to finding special instances that differ from given normal instances. Conventional computer vision-based anomaly detection mainly adopts image processing methods [23,24] and machine learning methods that are based on hand-crafted feature extraction [25,26]. However, image processing methods are non-learning methods that do not utilize existing data. The performance of machine learning methods mainly depends on the quality of the hand-crafted features and few features are specially designed for anomaly detection, so it is difficult to obtain satisfactory results. Recently, deep learning-based anomaly detection methods have received extensive attention. There are many existing methods for industrial product anomaly detection [8,27,28]. Recent studies have shown that image reconstruction-based methods can be effective in addressing the problem of the lack of defective samples. However, most image reconstruction-based methods are only trained on easily accessible defect-free images. Schlegl et al. [29] proposed an anomaly detection method that was based on a vanilla generative adversarial network (GAN), which captured the manifold of normal images and reconstructed the pseudo-images that were closest to the distribution of the normal images. Soukup et al. [30] proposed an autoencoder (AE)-based network that mapped images on to latent spaces through an encoder network and created reconstructed images that were similar to the input images using a decoder network. In the test phases, the above two methods used the differences between the query image and the reconstructed image to detect surface defects.

Although both the GAN-based method and AE-based method could solve the problem of the small number of defective samples, the GAN training requires expensive computational resources and the reconstructed images from the AE differ greatly from the input images, resulting in low defect detection accuracy. To overcome these problems, this paper addresses the challenge from three perspectives. First, in order to quickly match the latent vectors that were closest to the normal image distribution, we reconstructed the defect-free images using an encoder–decoder network, thereby avoiding the process of updating the input vectors to capture the normal image manifold. Second, to further enhance the representation ability of the extracted features, a novel attention mechanism that utilizes the parallel fusion of channel attention and pixel attention was added to the encoder network to enhance the detailed information attention of the network. Third, the latent vector that was extracted by the network only contained the features of the normal samples and it was not clear whether the features of abnormal samples were also learned. Therefore, this study proposed a new consistency loss function that was based on the pixel consistency, structural consistency and gradient consistency of the images to further improve the ability of the network to reconstruct normal samples, inhibit abnormal reconstruction and improve the accuracy of the detection of defective samples.

To summarize, the contributions of this paper are threefold:

- An encoder–decoder generative adversarial network is proposed that directly maps image spaces on to latent spaces;
- A novel dual attention block is proposed within the encoder network;
- A consistency loss function is proposed to enhance the ability of the network to reconstruct defect-free images.

This paper is organized as follows. Section 2 presents related work on surface defect detection and anomaly detection. Section 3 introduces our proposed network structure and the training strategy for our dual attention-based industrial surface defect detection method with consistency loss. Section 4 describes the dataset that we used, the training details and the experimental results. In Section 5, we draw conclusions through experiments.

## 2. Related Work

At present, surface defects on industrial products seriously affect the quality and efficiency of production and a number of industrial enterprises have introduced products that are related to the detection of surface defects. Cognex’s deep learning defect detection tool can learn to find a variety of unacceptable product defects throughout the manufacturing process. This tool inspects the screen, band and back of a smartphone before it is packaged. It is used to detect any combination of dents, scratches and discolorations anywhere on the smartphone. Zeiss proposed SurfMax, which obtains three different modes of captured images (grayscale images, gloss images and slope images) based on deflection measurements using a high-resolution Zeiss optical sensor. It completely captures the relevant surface features and is then combined with machine learning methods to carry out surface defect detection in automotive, aerospace, medical and consumer electronics manufacturing. Creaform designed 3D scanners for the non-destructive inspection of gas pipelines and aerospace surfaces. Therefore, surface defect detection has become a research hot spot for some companies at present. In this section, we summarize the related work within surface defect detection and anomaly detection.

### 2.1. Surface Defect Detection

According to the extracted features and detection algorithms, the traditional surface defect detection methods within image processing can be divided into three categories: the statistical method [31], frequency spectrum method [32] and model method [33]. The traditional methods are no longer applicable due to their high human costs and their inability to represent high-dimensional data features. The rapid development of deep learning within the field of computer vision, especially the strong feature extraction ability of deep networks, has opened up new possibilities for industrial surface defect detection [34,35].

Industrial surface defect detection can improve the qualified rate and overall quality of products and it is used in a variety of tasks. Therefore, many defect detection algorithms have been proposed [36,37,38,39]. Due to the variety of defective samples and the difficulty in collecting them, most of the current surface defect detection methods are based on unsupervised or semi-supervised image reconstruction methods that rely on reconstruction errors or other measurement methods (such as latent vector errors, etc.) to detect defects. The ultimate goal of the AE-based method is to enable the encoder to learn the good low-dimensional features of a normal input image and to reconstruct the input image. Youkachen et al. [40] used a convolutional autoencoder (CAE) to reconstruct an image and complete the surface defect segmentation of a hot rolled strip. Their final surface defect segmentation results were obtained from the reconstruction error, following a sharpening treatment. Bergmann et al. [41] believe that the $l^{p}$ distance measure between pixels could lead to large residuals in the reconstruction of image edges, so they added the structural similarity (SSIM) measure to the loss function. Their results showed that the detection performance was significantly improved compared to the per-pixel reconstruction error metric.

### 2.2. Anomaly Detection

Anomaly detection, also known as outlier detection or novel detection [42], refers to the process in which detected data deviate significantly from normal data. In surface defect detection, defects in images can be regarded as abnormal instances, so we can apply anomaly detection to find defect images. The experiments of a large number of researchers have shown that using anomaly detection methods to detect defects is effective. Nakanishi et al. [43] considered the insufficient reconstruction accuracy of many of the AE-based methods. Natural images are mostly low frequency, so they introduced a weighted frequency domain loss (WFDL) from the perspective of the frequency domain to improve the reconstruction of high-frequency components, which made the reconstructed images clearer and improved the accuracy of the anomaly detection. Recently, many researchers have completed anomaly detection tasks using the GAN-based method [27,29,44]. The ultimate goal of the GAN-based method is to enable the generator to learn the intrinsic laws of normal samples and create reconstructed images that are similar to the normal images using the learned knowledge. In order to reduce computing resources, Akcay et al. proposed the GANomaly [28] network to reconstruct images by encoding and decoding the input image without the need to iteratively search for the latent vectors. They defined the anomaly score by encoding the latent vectors of input images and reconstructed images. Inspired by the skip connection structure of U-Net [45], Akcay et al. proposed skip-GANomaly [46], which has a stronger image reconstruction power than GANomaly. The anomaly score emphasizes the differences between the reconstructed and input images, but this method still has the problem of inaccurate detection. Tang et al. proposed a dual autoencoder GAN (DAGAN) [47], which combined the ideas of BEGAN [48] and skip-GANomaly. The generator and discriminator were composed of two autoencoders to improve the image reconstruction ability and training stability. Carrara et al. proposed CBiGAN [49], which introduced a consistency constraint regularization term into the encoder and decoder of BiGAN [50] to improve the quality and accuracy of the image reconstruction.

## 3. Proposed Method

**Figure 2:**
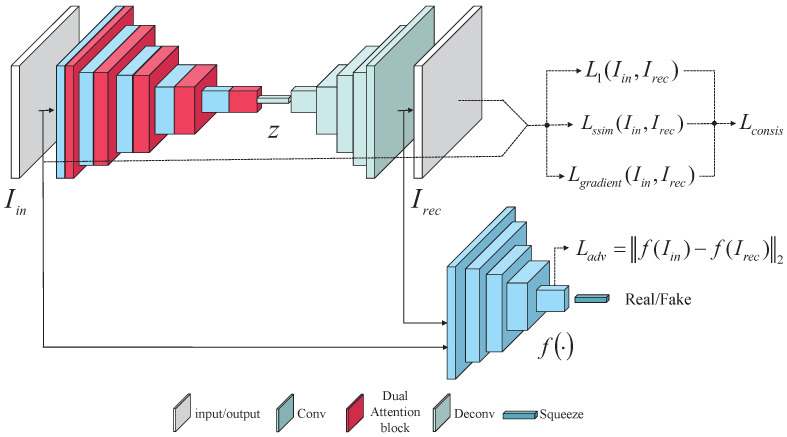
The network architecture of the proposed method.

In this section, we first introduce the proposed framework for the detection of industrial surface defects (as shown in Figure 2). Then, we describe in detail the dual attention module structure that we embedded into the generative network, as well as the discriminative network structure. Next, the training strategy that we employed to train our model using normal images is introduced. Finally, we define the method that we used to calculate the anomaly scores for our defect detection.

### 3.1. Network Architecture

#### 3.1.1. Generative Network

As shown in Figure 2, the proposed generative network was based on the autoencoder structure, which is mainly composed of an encoder $G_{E}$ and a decoder $G_{D}$. The encoder network consisted of a convolutional layer, a dual attention block and a batch normalization layer. The decoder network was composed of a deconvolutional layer and a batch normalization layer. The goal of the generative network was to reconstruct the image that was closest to the defect-free input image. The input image first entered the encoder network, which acted as the feature extraction process by mapping the image on to the latent space. The encoding process could be represented as:

(1) $$z=f_{e}\left(I_{in}\right)$$

where *z* represents the feature vector in the latent space, $f_{e}$ represents the encoding process and $I_{in}$ is the input image.

The latent vectors were then decoded by the network and reconstructed in the image space. The decoding procedure could be expressed as:

(2) $$I_{rec}=f_{d}\left(z\right)$$

where $I_{rec}$ is the reconstructed image and $f_{d}$ represents the decoding process.

#### 3.1.2. Dual Attention Block

**Figure 3:**
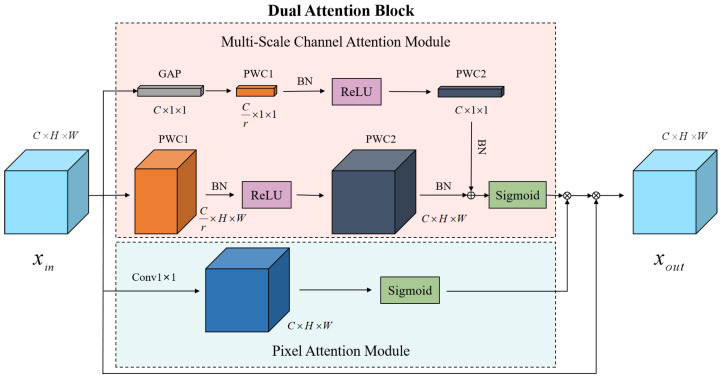
The dual attention block: the parallel fusion of multi-scale channel attention and pixel attention. ⊕ denotes the broadcasting addition and ⊗ denotes the element-wise multiplication.

In order to improve the quality of the network reconstruction of normal images, inspired by the methods that were proposed by Zhao et al. [51] and Dai et al. [52], we combined a pixel attention module (PAM) and a multi-scale channel attention module (MS-CAM) in the encoder network to form a dual attention block, which was connected to the convolutional layer. The dual attention block is shown in Figure 3.

We fused multi-scale channel attention and pixel attention in parallel. By using MS-CAM to enhance the network’s attention to image channel information, varying the size of the spatial pooling allowed for channel attention at multiple scales. First of all, the channel attention of global features performed a global averaging pooling (GAP) operation on the feature map $x_{in}$ to obtain $x_{1}=GAP\left(x_{in}\right)$ and then used a kernel size of $\frac{C}{r}\times C\times 1\times 1$ for point-wise convolution (PWC1, where r>1) to extract the features $x_{2}\;=\;PWC1\left(x_{1}\right)$. After processing with the batch normalization (BN) layer and ReLU activation function, $x_{3}=ReLU\left(BN\left(x_{2}\right)\right)$ was obtained using a kernel size of $C\times \frac{C}{r}\times 1\times 1$ for the point-wise convolution (PWC2) operation and the feature map $x_{4}$ was obtained from the BN layer $x_{4}=BN\left(PWC2\left(x_{3}\right)\right)$. The channel attention of local features also used PWC1 with a kernel size of $\frac{C}{r}\times C\times 1\times 1$ and PWC2 with a kernel size of $C\times \frac{C}{r}\times 1\times 1$ to extract features that were different from the channel attention for the global features. No global average pooling operation of the feature map was performed and the feature map $x_{5}$ was obtained from the channel attention of the local features. The feature map $x_{4}$ was broadcast into C×H×W dimensions and then added pixel by pixel to the feature map $x_{5}$ to obtain a more comprehensive focus on the feature information. The Sigmoid activation function was used to obtain the attention map $x_{6}$, $x_{6}=\delta \left(x_{4}⊕x_{5}\right)$ (δ denotes the Sigmoid activation function and ⊕ denotes the broadcasting addition). Then, the pixel attention module paid more attention to the information of each pixel within the image so it could generate a 3D (C×H×W) attention feature matrix, which used a 1×1 convolution kernel to perform the convolution operations on the feature map of the previous layer and used the convolution results to obtain the attention map $x_{7}$ using the Sigmoid activation function $x_{7}=\delta \left(Conv_{1}\left(x_{in}\right)\right)$. Finally, the attention map was obtained using parallel MS-CAM and PAM and the results were multiplied pixel by pixel to create the final attention feature map $x_{out}$, $x_{out}=x_{in}⊗\left(x_{6}⊗x_{7}\right)$ (⊗ denotes the element-wise multiplication).

#### 3.1.3. Discriminative Network

The discriminative network consisted of a convolutional layer and a batch normalization layer. The network received the real input image and the corresponding reconstructed image and then output a scalar value. After the Sigmoid function operation, the scalar value range was limited to between 0 and 1. The discriminator output a large scalar value (close to 1) for the real input image and a small scalar value (close to 0) for the reconstructed image. As the reconstructed image became more and more realistic after reaching the Nash equilibrium [53], the reconstructed image became realistic enough to deceive the discriminator. The output of the discriminative network could be represented as:

(3) $$D_{s}=f_{dis}\left(I_{query}\right)$$

where $D_{s}$ represents the output of the discriminative network, $f_{dis}$ represents the discriminant process and $I_{query}$ is the query image. The query image could be either the real input image or the corresponding reconstructed image.

### 3.2. Training Strategy

In the training phase, the encoder performed feature extraction on the input defect-free image and mapped the image on to the latent space. The decoder then reconstructed the extracted latent feature vectors into a pseudo-image. The discriminator distinguished between the input image and the pseudo-image and output a discriminant score, which eventually caused the pseudo-image that was reconstructed by the decoder to become infinitely closer to the input image.

The training process of the entire network could be described as follows:

- First, the generative network weights and the discriminative network weights were initialized, then the generative network weights were fixed and the discriminative network weights were updated. The discriminative loss adopted the binary classification cross-entropy loss within the classical GAN;
- After the discriminative network weights were updated, the discriminative network weights were fixed and the generative network weights were updated. Adversarial loss and consistency loss were introduced when updating the generative network weights.

The adversarial loss reduced the GAN’s training instability through feature matching. The $L^{2}$ distance in the middle-layer feature representations of the input and reconstructed images was employed as the loss function of the discriminator, which was expressed as follows:

(4) $$L_{adv}={∥f\left(I_{in}\right)-f\left(I_{rec}\right)∥}_{2}$$

To enhance the retention of the pixel and detailed information in the input image, the introduced consistency loss considered not only the pixels, but also the structural consistency and gradient consistency between the input image and the reconstructed image. The pixel consistency exploited the differences between the pixels in the input image and the reconstructed image to improve the image reconstruction ability. The structural consistency used SSIM to compare the real input image to the reconstructed image in terms of brightness, contrast and structure. The gradient of the image could reflect the frequency of image changes and improve the reconstruction quality of the high-frequency parts of the image. The consistency loss could be defined as:

(5) $$L_{consis}=L_{1}\left(I_{in},I_{rec}\right)+L_{ssim}\left(I_{in},I_{rec}\right)+L_{gradient}\left(I_{in},I_{rec}\right)$$

where $I_{in}$ represents the input image, $I_{rec}$ represents the reconstructed image and $L_{1}\left(I_{in},I_{rec}\right)={∥I_{in}-I_{rec}∥}_{1}$, ${∥\cdot ∥}_{1}$ represents the $L^{1}$ norm. A larger value of SSIM indicated a higher similarity between the two images, so it could be used as $L_{ssim}\left(I_{in},I_{rec}\right)=1-SSIM\left(I_{in},I_{rec}\right)$ to compute $L_{gradient}\left(I_{in},I_{rec}\right)={∥\nabla I_{in}-\nabla I_{rec}∥}_{1}$, where ∇ represents the gradient operations.

The adversarial loss and consistency loss were combined to update the total loss of the generative network parameters, which was indicated as:

(6) $$L_{total}=\alpha_{1}L_{adv}+\alpha_{2}L_{consis}$$

where $\alpha_{1}$ and $\alpha_{2}$ indicate the weight coefficients of the adversarial loss and the consistency loss, respectively.

In the testing phase, since the network could only reconstruct defect-free images, normal images were reconstructed from unseen defect images. Therefore, the inputs and outputs of defect images were quite different, especially around the defective areas, so the anomaly score could be obtained through using the discriminative network.

### 3.3. Anomaly Score

Assuming that the trained generative network was good enough to reconstruct defect-free images, we used the absolute value of the pixel-by-pixel difference between the query image and the reconstructed image as the anomaly score. Given the different thresholds for the different datasets, the anomaly score was determined as an anomaly when it was greater than the relevant threshold. The anomaly score was defined as:

(7) $$S^{\left(i\right)}=\left|I_{query}^{\left(i\right)}-I_{rec}^{\left(i\right)}\right|$$

where $I_{query}^{\left(i\right)}$ and $I_{rec}^{\left(i\right)}$ are the $i_{th}$ query image and the reconstructed image, respectively, and |·| is the absolute value operation.

Using Equation (7), we were able to calculate the anomaly score for each query image. The anomaly scores of all of the query images formed an anomaly score vector of *S*, which was restricted to [0,1] by feature scaling. The final anomaly score could be expressed as:

(8) $$S_{i}=\frac{S^{\left(i\right)}-S_{\min}}{S_{\max}-S_{\min}}$$

where $S_{\max}$ and $S_{\min}$ represent the maximum and minimum values of the vector *S*, respectively.

## 4. Experiments

In this section, we evaluate the proposed method in terms of the surface defect detection problem. We first present the datasets that were used, followed by a discussion of some of the training details and evaluation metrics that were used in the experiments. Finally, we compare our method to several existing defect detection algorithms. Using the MVTec AD dataset [54], we compared the AnoGAN [29], GANomaly [28], skip-GANomaly [46], DAGAN [47] and CBiGAN [49] algorithms. Using the Magnetic Tile dataset [55], we compared the GANomaly and Adgan [27] algorithms.

### 4.1. Datasets

This experiment used the MVTec AD dataset [54] and the Magnetic Tile dataset [55] for the defect detection.

MVTec AD is a real-world dataset of industrial surface defects with 5354 high-resolution images. The dataset contains 15 different industrial product surfaces, each of which is divided into a training set and a testing set. The training set only contains defect-free images, while the testing set contains both defect-free images and 70 types of defect images. The details of the MVTec AD dataset are shown in Table 1.

**Figure 4:**
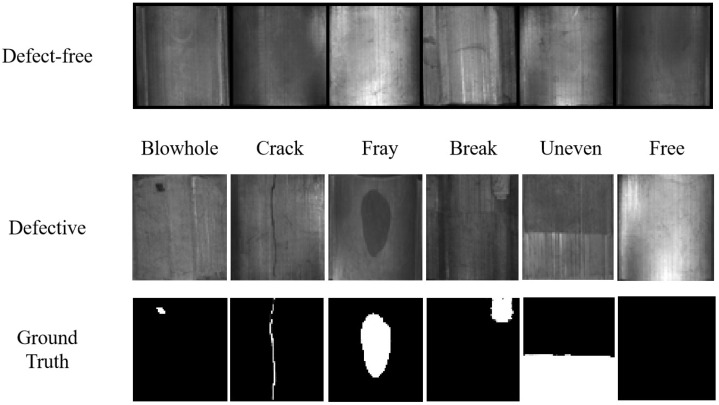
The defect-free and defect samples in the Magnetic Tile dataset.

The Magnetic Tile dataset has 1344 grayscale images under multiple illumination conditions, including 952 defect-free images. We randomly selected 80% as the training set and the remaining defect-free images and 392 defect images were merged together as the testing set, which included six defect types: blowhole, crack, fray, break, uneven and free. All of the images had pixel-level labels, as shown in Figure 4.

### 4.2. Training Details

**Figure 5:**
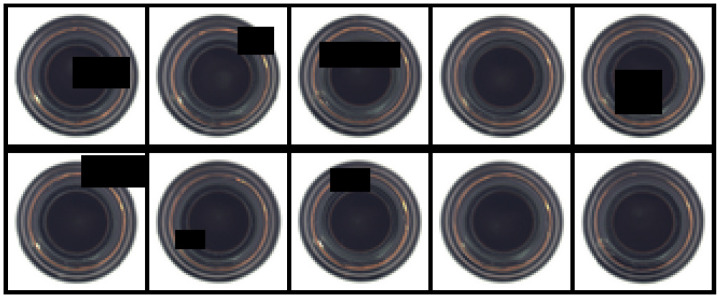
The Random Erasing data enhancement processing.

To enhance the robustness of the generative network for defect image reconstruction, we used Random Erasing [56] data enhancement processing for the training set, with the Random Erasing probability set to 0.3. The data enhancement is shown in Figure 5. In addition, considering the different resolutions of the images in each dataset, we resized the input images to 256×256. In particular, images from the MVTec AD dataset employed 3-channel images as the input, while the Magnetic Tile dataset employed single-channel images.

For all of the experiments in this paper, we employed five convolutional layers and used a dual attention block after each layer as the encoder network. The latent vectors were reconstructed after five deconvolutional layers. For the training of the generator and discriminator networks, we set the batch size to 64, used Adam [57] as the optimizer with a learning rate of $1\times 10^{-4}$ and set the momentum parameters as $\beta_{1}=0.5,\beta_{2}=0.999$. The weights for the total loss $L_{total}$ were set to $\alpha_{1}=1$ and $\alpha_{2}=40$. All of the experiments in this paper used Pytorch 1.8.0, CUDA 11.1 and CUDNN 8.0.5. All of the experiments were performed on a computer with an Intel Core i9-10900K CPU, 64GB RAM and NVIDIA GeForce RTX 3090 GPU.

### 4.3. Evaluation Indicators

To evaluate the performance of the proposed method for defect detection, the AUC [58] value was utilized (the area under the curve of the receiver operating characteristics (ROC)), which had a true positive rate on the horizontal axis and a false positive rate on the vertical axis.

### 4.4. Experimental Results

In this subsection, we compare several popular reconstruction-based defect detection methods to verify the superiority of the proposed method for the surface defect detection problem.

First, we compared the performance of the proposed method to several other defect detection methods: AnoGAN [29], GANomaly [28], skip-GANomaly [46], DAGAN [47] and CBiGAN [49]. Among them, AnoGAN generates pseudo-images that are similar to the probability distribution of the normal samples using random noise and the anomaly score consists of the difference between the pixel space of the input image and that of the generated image and the difference between the feature maps of the last layer of the discriminator network. GANomaly reconstructs images using an encoder–decoder–encoder process and defines the anomaly score by encoding the differences between the input image and the generated image to obtain a latent vector. Inspired by the skip connection structure, skip-GANomaly improves the structure of GANomaly to obtain a stronger image reconstruction ability and the anomaly score emphasizes the differences between the reconstructed image and the input image. The generator and discriminator networks of DAGAN are composed of two autoencoders, which improves the network’s ability to reconstruct images and its training stability. CBiGAN introduces a consistency-constrained regularization term within the encoder and decoder, resulting in an improved reconstruction accuracy. The comparison results are shown in Table 2. From the results, it can be observed that our method achieved the best performance using the MVTec AD dataset. Although skip-GANomaly and DAGAN demonstrated a strong reconstruction ability, they do not have attention mechanisms added into their networks, which resulted in a lack of attention to detail in the images. Our model showed a more comprehensive feature extraction ability due to the addition of the dual attention block, which provided a further supplement to the detailed features. As can be seen from Table 2, the defect detection performance of our method was greatly improved compared to the other methods. Our results for the cable and pill categories were 7% and 8% higher than CBiGAN, respectively. For the capsule category, our result was 13% higher than GANomaly. For the carpet, leather, toothbrush and zipper categories, our results were 1%, 1%, 5% and 13% higher than DAGAN, respectively. For the transistor category, our result was 7% higher than skip-GANomaly. From the mean experimental results of the 15 categories, our method outperformed the existing methods by 3.3% and achieved the best results.

**Figure 6:**
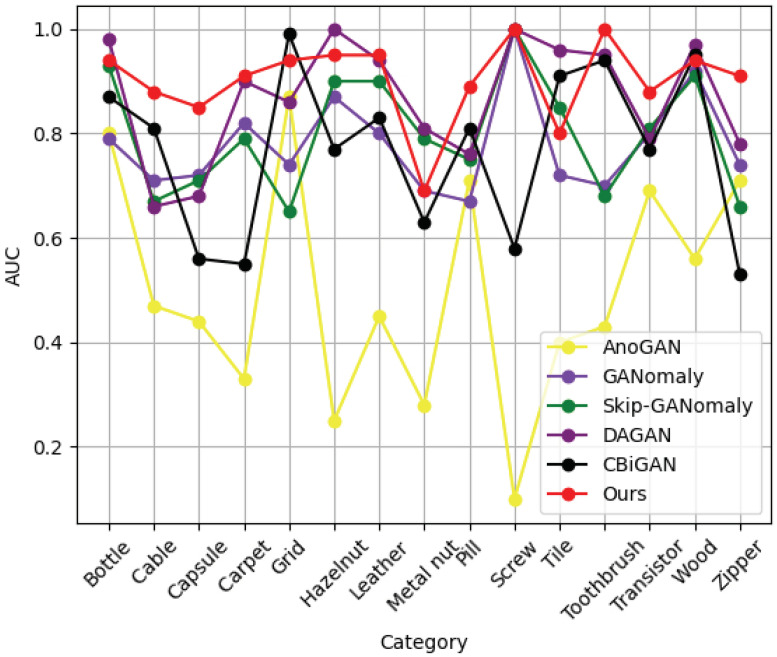
The AUC of our proposed method and five other GAN-based methods, which were tested using the MVTec AD dataset.

To highlight the superiority of the proposed method, we plotted an AUC line chart for each category of the MVTec AD dataset (as shown in Figure 6). It can be seen more intuitively from the figure that our proposed method showed a more robust performance for industrial defect detection than the other GAN-based methods. On the other hand, the line chart fluctuations for AnoGAN were large because it needed to iteratively search for the appropriate latent vectors, resulting in unstable training.

Then, the proposed method was further verified using the Magnetic Tile dataset. Compared to GANomaly and Adgan [27] (Adgan uses a scalable encoder–decoder–encoder architecture), fine-grained reconstructed images of normal classes could be obtained by extracting and exploiting the multi-scale features of normal samples. The comparison results are shown in Table 3. The images from the Magnetic Tiles dataset are under different illumination conditions that have a great impact on defect detection, so the two methods showed poor defect detection performances. Our proposed consistency loss enhanced the sensitivity of our model under different illumination conditions and improved the defect detection ability. From the experimental results, it can be observed that our model could also be trained stably using grayscale images under varying illumination conditions, with an 8% and 38% improvement over GANomaly and Adgan, respectively. GANomaly and Adgan have similar encoder–decoder–encoder structures and have good reconstruction abilities under simple conditions, but for complex scenes, their defect detection performances were poor. We comprehensively considered the characteristics of image pixels, structure and gradient so that our model could maintain a good reconstruction ability for complex scenes and obtain an excellent defect detection ability.

**Figure 7:**
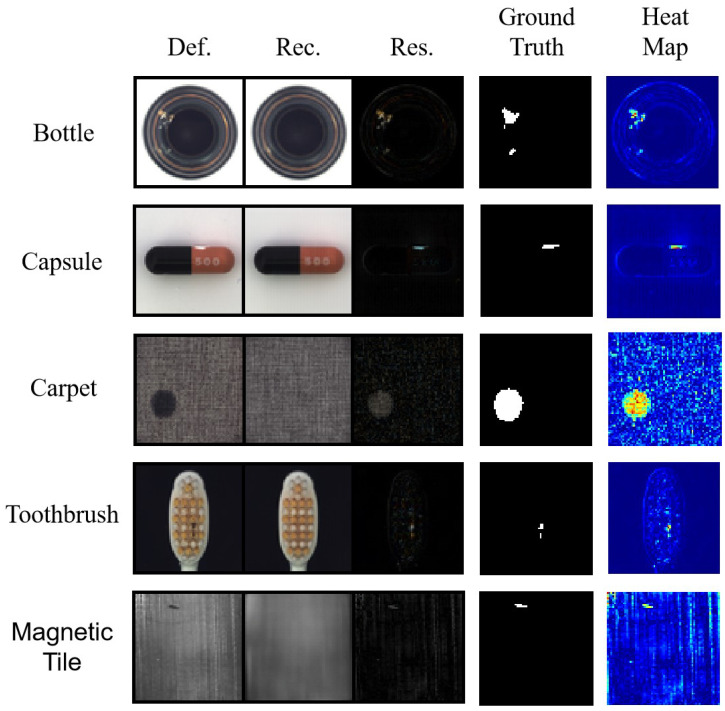
The first four rows of images show the test results from the partial MVTec AD dataset and the fifth row shows the test results from the Magnetic Tile dataset. Def. represents the defect image, Rec. represents the reconstructed image and Res. represents the residual image.

The results of the sample study using the MVTec AD and Magnetic Tile datasets are shown in Figure 7. This figure shows that our proposed method could reconstruct defect images as defect-free images. Using the residual map of the defect image and the reconstructed image, the defect could be found easily. By comparing the heat maps and ground truths, it can be observed that our model could accurately detect the location of defects.

### 4.5. Ablation Studies

In this subsection, we present the results from our ablation studies, in which we performed a group of experiments to verify the effectiveness of the individual strategies within our proposed model, mainly from two perspectives: the effectiveness of the dual attention block and the effectiveness of the consistency loss.

#### 4.5.1. Effectiveness of the Dual Attention Block

**Figure 8:**
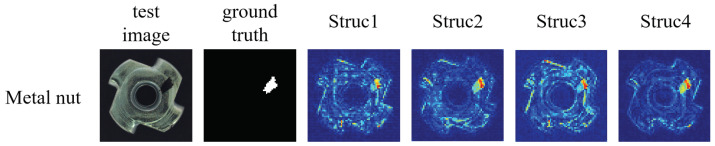
The heat maps of an image of a defective metal nut using the different attention structures.

We fixed our proposed consistency loss as the loss function of the generative network by changing the attention in the encoder network and constructing four different structures. First, the dual attention block was removed from the encoder network to evaluate the defect detection performance without the attention mechanism, which was named **Struc1**. Multi-scale channel attention was introduced into the encoder network to evaluate the effects of channel attention on the defect detection performance, which was named **Struc2**. Then, the channel attention in the encoder network was replaced with pixel attention to evaluate the impacts of pixel attention on the defect detection, which was named **Struc3**. Finally, our proposed method was named **Struc4**. As can be seen from Table 4 and Table 5, the different structures achieved different results by adjusting the attention mechanism, although **Struc2** and **Struc3** had higher AUC values for some categories in the MVTec AD dataset, the encoder network for the parallel fusion of multi-scale channel attention and pixel attention could effectively extract the key information so **Struc4** achieved a mean AUC value of 90.2%. Using the Magnetic Tile dataset, we counted the running speed that was needed to train and test each image for the different structures. Although **Struc4** took a little longer than the other structures, it achieved the highest result of 84%. As shown in Figure 8, we used the heat map of the metal nut category to test the ability of the four structures to detect defects in the same image. By comparison, **Struc4** had less noise in the defect heat map and could detect defects more accurately.

#### 4.5.2. Effectiveness of the Consistency Loss

Without changing the dual attention network structure, we considered the loss function in the network in three ways. First, the loss function in the generative network used the pixel consistency loss function **$L_{1}$** to evaluate its impact on the image reconstruction ability. Second, based on the pixel consistency loss function, the structural consistency loss function was added and the two functions were used as the generative network loss function to evaluate the generation effects, namely **$L_{1}+L_{ssim}$**. Third, we combined pixel consistency, structural consistency and gradient consistency to constitute the consistency loss function, namely **$L_{1}+L_{ssim}+L_{gradient}$**. The experimental results are shown in Table 6 and Table 7. The results show that the proposed consistency loss achieved the best performance using the two datasets.

## 5. Conclusions

We studied the problem of detecting surface defects during the production process of industrial products, i.e., the wide variety of surface defects among different products and the difficulty in collecting defective samples. Therefore, we proposed a semi-supervised anomaly detection method that was based on dual attention and consistency loss to accomplish this task. We used an encoder–decoder structure for the generative network and introduced a dual attention module into the encoder network, which combined multi-scale channel attention and pixel attention. The parallel fusion of the two kinds of attention mechanism improved the performance of key feature extraction and reconstructed higher quality defect-free images. In addition, the consistency loss made use of the differences between the pixels, structures and gradients of the defect images and defect-free images to further improve the performance of the defect detection. Comprehensive experiments using the MVTec AD and Magnetic Tile datasets showed that the proposed method could achieve a superior performance over the existing methods.

## Figures and Tables

**Table 1:** The MVTec AD dataset: N represents a defect-free sample and P represents a defect sample.

Category	Training Set (N)	Testing Set (N)	Testing Set (P)	Resolution
Bottle	209	20	63	900×900
Cable	224	58	92	1024×1024
Capsule	219	23	109	1000×1000
Carpet	280	28	89	1024×1024
Grid	264	21	57	1024×1024
Hazelnut	391	40	70	1024×1024
Leather	245	32	92	1024×1024
Metal Nut	220	22	93	700×700
Pill	267	26	141	800×800
Screw	320	41	119	1024×1024
Tile	230	33	84	840×840
Toothbrush	60	12	30	1024×1024
Transistor	213	60	40	1024×1024
Wood	247	19	60	1024×1024
Zipper	240	32	119	1024×1024

**Table 2:** The area under the receiver operating characteristic curve of the MVTec AD dataset. The results in bold were the best AUC results during the tests and the underlined results were the suboptimal AUC results.

Category	AnoGAN	GANomaly	Skip-GANomaly	DAGAN	CBiGAN	Ours
Bottle	0.80	0.79	0.93	**0.98**	0.87	0.94
Cable	0.47	0.71	0.67	0.66	0.81	**0.88**
Capsule	0.44	0.72	0.71	0.68	0.56	**0.85**
Carpet	0.33	0.82	0.79	0.90	0.55	**0.91**
Grid	0.87	0.74	0.65	0.86	**0.99**	0.94
Hazelnut	0.25	0.87	0.90	**1.00**	0.77	0.95
Leather	0.45	0.80	0.90	0.94	0.83	**0.95**
Metal Nut	0.28	0.69	0.79	**0.81**	0.63	0.69
Pill	0.71	0.67	0.75	0.76	0.81	**0.89**
Screw	0.10	**1.00**	**1.00**	**1.00**	0.58	**1.00**
Tile	0.40	0.72	0.85	**0.96**	0.91	0.80
Toothbrush	0.43	0.70	0.68	0.95	0.94	**1.00**
Transistor	0.69	0.80	0.81	0.79	0.77	**0.88**
Wood	0.56	0.92	0.91	**0.97**	0.95	0.94
Zipper	0.71	0.74	0.66	0.78	0.53	**0.91**
**Mean**	0.499	0.779	0.800	0.869	0.766	**0.902**

**Table 3:** The area under the receiver operating characteristic curve of the Magnetic Tile dataset. Bold number represents the optimal result.

Method	GANomaly	Adgan	Ours
AUC	0.76	0.46	**0.84**

**Table 4:** The test results from the different attention structures using the MVTec AD dataset. Bold number represents the optimal result.

Category	Struc1	Struc2	Struc3	Struc4
Bottle	0.95	0.94	**0.96**	0.95
Cable	**0.90**	0.87	0.87	0.88
Capsule	0.79	**0.85**	**0.85**	**0.85**
Carpet	0.83	**0.91**	0.88	**0.91**
Grid	0.87	0.87	0.92	**0.94**
Hazelnut	0.94	**0.97**	0.92	0.95
Leather	0.89	0.89	**0.95**	**0.95**
Metal Nut	0.63	0.64	0.62	**0.69**
Pill	0.86	0.87	**0.89**	**0.89**
Screw	**1.00**	**1.00**	**1.00**	**1.00**
Tile	0.71	0.70	0.71	**0.80**
Toothbrush	**1.00**	**1.00**	0.99	**1.00**
Transistor	0.86	0.87	0.86	**0.88**
Wood	**0.94**	0.93	**0.94**	**0.94**
Zipper	**0.91**	0.88	0.89	**0.91**
**Mean**	0.872	0.879	0.884	**0.902**

**Table 5:** The AUC results from the different attention structures, which were tested using the Magnetic Tile dataset, and the running speeds (in seconds), which were measured for each image during training and testing. Bold number represents the optimal result.

Method	Struc1	Struc2	Struc3	Struc4
AUC	0.75	0.82	0.79	**0.84**
Training Speed (s)	0.0931	0.1550	0.1026	0.1557
Testing Speed (s)	0.0276	0.0487	0.0413	0.0511

**Table 6:** The AUC values from the different loss functions using the MVTec AD dataset. Bold number represents the optimal result.

Category	$L_{1}$	$L_{1}+L_{\mathrm{ssim}}$	$L_{1}+L_{\mathrm{ssim}}+L_{\mathrm{gradient}}$
Bottle	**0.95**	0.94	0.94
Cable	0.82	0.86	**0.88**
Capsule	**0.85**	**0.85**	**0.85**
Carpet	0.88	**0.92**	0.91
Grid	0.87	0.86	**0.94**
Hazelnut	**0.96**	0.94	0.95
Leather	0.91	0.88	**0.95**
Metal Nut	0.62	0.62	**0.69**
Pill	0.86	**0.90**	0.89
Screw	**1.00**	**1.00**	**1.00**
Tile	0.73	0.72	**0.80**
Toothbrush	**1.00**	**1.00**	**1.00**
Transistor	0.87	0.87	**0.88**
Wood	**0.94**	0.93	**0.94**
Zipper	0.89	0.90	**0.91**
**Mean**	0.876	0.879	**0.902**

**Table 7:** The AUC values from the different loss functions using the Magnetic Tile dataset. Bold number represents the optimal result.

Method	$L_{1}$	$L_{1}+L_{\mathrm{ssim}}$	$L_{1}+L_{\mathrm{ssim}}+L_{\mathrm{gradient}}$
AUC	0.82	0.83	**0.84**

## Data Availability

Data sharing not applicable.
